# Region Specific Response of Intervertebral Disc Cells to Complex Dynamic Loading: An Organ Culture Study Using a Dynamic Torsion-Compression Bioreactor

**DOI:** 10.1371/journal.pone.0072489

**Published:** 2013-08-28

**Authors:** Samantha C. W. Chan, Jochen Walser, Patrick Käppeli, Mohammad Javad Shamsollahi, Stephen J. Ferguson, Benjamin Gantenbein-Ritter

**Affiliations:** 1 Tissue and Organ Mechanobiology group, Institute for Surgical Technology & Biomechanics, University of Bern, Bern, Switzerland; 2 Institute for Biomechanics, ETH Zürich, Zürich, Switzerland; University of Utah, United States of America

## Abstract

The spine is routinely subjected to repetitive complex loading consisting of axial compression, torsion, flexion and extension. Mechanical loading is one of the important causes of spinal diseases, including disc herniation and disc degeneration. It is known that static and dynamic compression can lead to progressive disc degeneration, but little is known about the mechanobiology of the disc subjected to combined dynamic compression and torsion. Therefore, the purpose of this study was to compare the mechanobiology of the intervertebral disc when subjected to combined dynamic compression and axial torsion or pure dynamic compression or axial torsion using organ culture. We applied four different loading modalities [1. control: no loading (NL), 2. cyclic compression (CC), 3. cyclic torsion (CT), and 4. combined cyclic compression and torsion (CCT)] on bovine caudal disc explants using our custom made dynamic loading bioreactor for disc organ culture. Loads were applied for 8 h/day and continued for 14 days, all at a physiological magnitude and frequency. Our results provided strong evidence that complex loading induced a stronger degree of disc degeneration compared to one degree of freedom loading. In the CCT group, less than 10% nucleus pulposus (NP) cells survived the 14 days of loading, while cell viabilities were maintained above 70% in the NP of all the other three groups and in the annulus fibrosus (AF) of all the groups. Gene expression analysis revealed a strong up-regulation in matrix genes and matrix remodeling genes in the AF of the CCT group. Cell apoptotic activity and glycosaminoglycan content were also quantified but there were no statistically significant differences found. Cell morphology in the NP of the CCT was changed, as shown by histological evaluation. Our results stress the importance of complex loading on the initiation and progression of disc degeneration.

## Introduction

Healthy intervertebral disc (IVD) function as essential mechanical load-transmitters between the vertebrae, allowing compression, bending, flexion and torsion of the spine. It is clear that some of the causes of low back pain, such as disc herniation and degeneration, are influenced by mechanical loading, which is related to the life style and daily activity of the individual [1]–[3]. Daily cyclic diurnal loading is important for disc health, as it assists in the transport of large soluble factors across the disc and from its surrounding vascular supply and applies a direct and indirect stimulus to disc cells. The intervertebral disc experiences various complex loading conditions of different magnitudes and directions consisting of compression, axial rotation, flexion and extension.

Epidemiological studies identified both compression and axial torsion as risk factors in the development of disc degeneration and back pain [4], [5]. Many studies have demonstrated that acute mechanical injury and accumulated overloading could induce disc degeneration [6], [7]. Compression is known to cause disc degeneration (DD) in a magnitude, frequency and duration dependent manner. Characteristics of DD include increased cell death, a decrease in disc height due to a loss of essential matrix components which can also be reflected by an increased matrix catabolic gene expression (MMP-3, MMP-13, ADAMTS-4) but decreased anabolic gene expression (collagens and proteoglycans), increased inflammatory response (TNF-a, IL-1b, IL-6) and changes of mechanical properties of the disc (increased stiffness) [8].

During the day, the disc experiences a pressure range from 0.1–1.1 MPa [9], [10]. However, studies have shown that dynamic compressive loading of >0.8 MPa could induce early DD [11]–[13]; dynamic loading of physiological magnitude (1 MPa) at a frequency of 0.2 Hz was suggested to be the best in preserving disc metabolism while a frequency of 0.01 or 1 Hz could stimulate catabolic gene expressions [14]; signs of mild disc degeneration were seen when loading was applied in a longer term of 8 weeks (8 h/day) even at a physiological magnitude (1 MPa) [15]. A recent study also demonstrated that the complex loading of side bending (in the form of asymmetric compression) and cyclic compression induced a greater structural disruption to the disc than simple cyclic compression [16].

The influence of compression on the disc has been well studied, but much less is known about the role of torsion in disc degeneration. Only recently, the role of torsion on the disc’s health has returned to focus, although Farfan and colleagues had investigated the relationship between torsion and disc degeneration biomechanically already in the 70 s [17]. Hadjipavlou et al have demonstrated, using an in-vivo rabbit model, that torsional injury is one of the initiators of disc degeneration, as evidenced by a decrease in disc height and a drop in disc proteoglycan content [18], [19]. The influence of torsion on the gene expression of the disc has been investigated in vivo in a rat animal model using an Ilizarov-type fixator implanted to the caudal motion segments to stimulate tail discs [20]. It was shown that cyclic torsion could cause injury to the disc, provoking increased inflammatory (TNF-α and IL-1β ) and altered elastin gene expressions [20]. An increase in elastin content in the AF is one of the observations in degenerated human discs [21] and an alteration in the elastin fiber network might render the AF more susceptible to micro failure under torsion and bending [22].

Complex loading consisting of compression, torsion or flexion/extension increases the chances of disc injury/herniation at a lower force magnitude as shown in various biomechanical studies [23]–[28]. According to findings from biomechanical studies, it is apparent that the presence of different types of loading would change the overall response of the disc to mechanical loading [29]. When the spinal motion segment is twisted at a fixed torsion angle, the ultimate strength of the spinal motion segment to compression was decreased and the susceptibility to disc injury was therefore increased [25], [30]. Torsional stiffness of the spinal segment also increases with axial load, and it was suggested that axial loading combined with torsional stress may result in annulus damage and in disc degeneration [31]. Moreover, excessive bending is known to cause disc injury including herniation [24], [32]. With highly repetitive flexion-extension, disc herniation may happen with modest levels of compression (867–1,472 N) without damage to the facet joints in porcine specimens [24]. Disc herniation was also induced in fewer loading cycles when torsion was added to a flexed motion [23]. Mechanobiological studies have also highlighted the influence of combined loading on disc health. Walter et al. have demonstrated that asymmetric dynamic compression (bending with compression) caused annulus fibrosus (AF) delamination and cell apoptosis [16]. However, to our best knowledge the approach to stimulate organ culture bovine disc explants in vitro under controlled two degree of freedom, dynamic compression and torsion, has not been investigated previously.

Studying the influence of dynamic torsion-compression loading on the disc will improve our understanding of the response of disc cells and matrix under a complex strain stimulus, as applied by torsion, and reveal possible etiology of DD related to torsion-compression loading. This study aimed to investigate the cellular responses of the disc when exposed to complex dynamic torsion-compression loading, as compared to single mode of dynamic torsion or compression. We hypothesized that complex dynamic torsion- compression loading induces high strains in the NP and AF and causes collagen and cell damage, hence increasing the susceptibility of the intervertebral disc to degeneration.

## Methods

### Bovine Intervertebral Disc Isolation and Organ Culture

**Figure 1:**
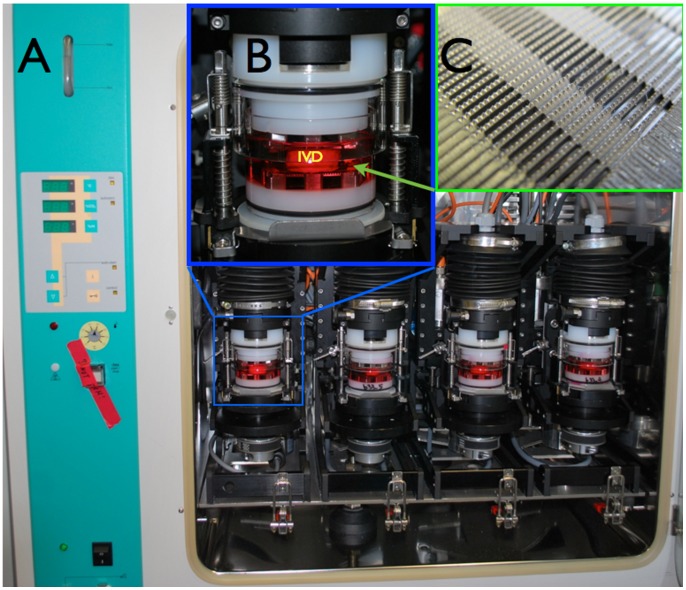
Detailed view of the loading bioreactor and specimen chamber. A) The bioreactor is situated inside a 37°C incubator with 5% CO_2_ and 80% humidity. B) The glass walled sample chamber. IVD: intervertebral disc. C) Enlarged view of the titanium plate for holding the samples for rotation movement.

A total of 40 bovine caudal discs with intact vertebral endplates were isolated from eight animals (five discs per animal, one was used for day 0 control, four were used for culturing under different loading modalities for 15 days). Samples from six animals were used for all molecular analysis and two animals were used for histological analysis. As previously described [33], bovine caudal discs with endplates were harvested from the tails of 18–24 month-old cows, obtained from a local slaughterhouse (animal samples and permission for using these animal parts for research obtained from a slaughterhouse, Schlachthof, Interlaken, Switzerland) within 3 h of death. The posterior parts of the discs were marked with a surgical pen for later proper alignment of the samples in the specimen-loading chamber. After separation from the vertebral bone, the bony endplate surfaces were jet-lavaged with Ringer’s balanced salt solution using a Pulsavac™ wound debridement irrigation system (Zimmer GmbH, Winterthur, Switzerland) [33]. Disc explants were placed in the center of the custom-made glass specimen chambers with the posterior side facing to the back of the bioreactor. The specimen chamber contained two serrated titanium plates for securing the disc samples during the culture and the load application (Figure 1B). The disc samples were maintained in 40 mL Dulbecco’s Modified Eagle’s Medium (DMEM, 4.5 g/L glucose) with 5% fetal calf serum (FCS), 100 U/mL penicillin, 100 µg/mL streptomycin (all from Gibco, Basel, Switzerland) and cultured at 37°C, 5% CO_2_, and 80% humidity for 15 days, refreshing the medium every 2–3 days.

### Disc Dimension Measurement

Disc diameter (d) and disc height (h) were measured using a digital caliper (Tesa, Renens, Switzerland) before the experiment to calculate the required force magnitude, and after the experiment to monitor changes in disc dimensions. Two diameters were measured in the annulus: at the observed largest width of the disc (d_1_) and the diameter perpendicular to the largest width (d_2_). To estimate the error of measuring the biological samples using the digital caliper, disc height and diameters of the same sample were measured for three times, and the percentage error was calculated from the standard error mean of the three measurements over the mean value. The intra-observer error was estimated to be 1.69% and 0.35% for diameter and height, respectively. To calculate the required force for load application, the disc was assumed to be an ellipse. The disc surface area (A) was calculated as
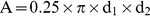
(1)


To calculate the disc volume before and after the loading, discs were assumed to be an elliptic cylinder and volume was calculated as
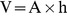
(2)


### Mechanical Loading

**Figure 2:**
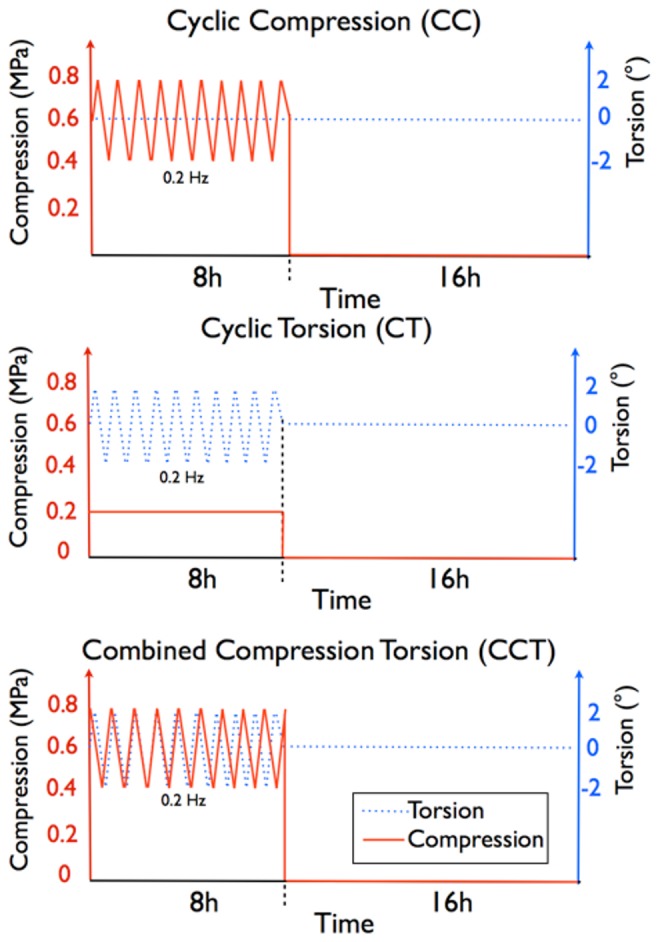
The 24-hour loading and resting cycles for the bovine organ culture. Top: cyclic compression (CC), middle: cyclic torsion (CT), bottom: combined compression & torsion (CCT). Samples were loaded for 8 h and then rested for 16 hours every day; loadings and resting were continued for 14 days.

The glass chambers containing the disc samples were fixed in a custom-built bioreactor for complex load application [34] (Figure 1). Discs from each tail of the same animal were assigned to 4 groups: 1) No loading (NL), 2) cyclic compression (CC), 3) cyclic torsion (CT), 4) combined compression torsion (CCT). In each group N = 6 samples were processed for molecular analysis and N = 2 for histological analysis. Except for NL, an 8 h of dynamic loading followed by a 16 h resting period were applied to the disc samples for 14 cycles, starting from the second day of culture. The 8 h dynamic loadings for each group were as follows: CC: 0.6 MPa ±0.2 MPa at 0.2 Hz, CT: ±2° rotation at 0.2 Hz and a static compression of 0.2 MPa, CCT: 0.6 MPa ±0.2 MPa at 0.2 Hz compression plus ±2° at 0.2 Hz rotation (Figure 2). The applied axial load was calculated from the disc endplate area, assuming a uniform pressure distribution within the disc. Discs in the NL group were cultured in the same glass chamber but no loading was applied during the 15 days of culture. After the final resting period on day 15, samples were dissected for analysis. Disc endplates were removed, NP and AF tissues were cut out using a 6 mm biopsy punch, and were further divided into 4 quarters. Each quarter was used for cell viability, metabolic activity, gene expression and caspase 3/7 activity analysis, respectively.

### Cell Viability

Cell viability of the tissues was evaluated with a LIVE/DEAD staining kit (Molecular Probes, Leiden, The Netherlands) as described before [35]. Briefly, NP and AF tissues (a quarter of a disc of 6 mm 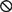 × 1 mm height) were incubated in 1 mL DMEM with 10 µmol/L calcein AM and 1 µmol/L ethidium homodimer-1 at 37°C for 3 h. Stained samples were visualized on an inverted confocal laser-scanning microscope (LSM510, Carl Zeiss, Germany). Cell viability was measured from three randomly chosen stacks. Stacks were split into single color channel images, and the number of green and red cells were counted using the nucleus counter plug-in from the McMaster Biophotonics Facility, http://www.macbiophotonics.ca/imagej/available from imageJ (http://imagej.nih.gov/ij/macros/Cellcounter3D.txt).

### Metabolic Activity

Cell activity of the disc tissue was measured using resazurin sodium salt assay (Sigma-Aldrich, Buchs, Switzerland). The dissected tissues were incubated in 500 µL DMEM containing 5% FCS and 50 µM resazurin sodium salt for 5 h in a 48-well plate. Relative fluorescence unit (RFU) was measured at an excitation wavelength of 547 nm and an emission wavelength of 582 nm using a microplate reader (SpectraMax M5, Molecular device, Switzerland). RFU measured for each tissue was normalized with the amount of DNA.

### Real-time Polymerase Chain Reaction

Disc tissues were flash-frozen in liquid N_2_ and then pulverized into powder with a mortar and pestle. The pulverized tissues were covered with 1 mL TRI reagent (Molecular Research Center, Cincinnati, OH, USA) and RNA was extracted using a modified TRIspin method [36]. RNA was purified using GenElute™ Mammalian total RNA purification kit with DNA digestion (Sigma-Aldrich, Buchs, Switzerland). Reverse transcription was performed (iScript cDNA Synthesis kit, Bio-Rad, Reinach, Switzerland). Bovine-specific oligonucleotide primers (Table 1) (Microsynth, Balgach, Switzerland) were newly designed with Beacon Designer™ software (Premier Biosoft, California, Palo Alto, USA) based on nucleotide sequences from GenBank. A cDNA template was mixed with the real-time reaction solution (IQ SYBR green supermix, Bio-Rad) and 0.25 µM primers. Real-time RT-PCR was run on an IQ5 cycler (Bio-Rad), followed by melting curve analysis. C_t_ values of the genes were normalized to the housekeeping gene ribosomal 18S RNA (ΔC_t_). ΔΔC_t_ values were calculated by normalizing the loading groups to the no loading control group and were then transformed to relative gene expression values by the 2^−ΔΔCt^-method [37].

**Table 1:** Bovine-specific oligonucleotide primers.

Gene	Forward	Reverse
18S	ACG GAC AGG ATT GAC AGA TTG	CCA GAG TCT CGT TCG TTA TCG
Aggrecan	GGC ATC GTG TTC CAT TAC AG	ACT CGT CCT TGT CTC CAT AG
Biglycan	CTG CCA CTG CCA TCT GAG	TTG TTC ACG AGG ACC AAG G
Collagen 1	GCC TCG CTC ACC AAC TTC	AGT AAC CAC TGC TCC ATT CTG
Collagen 2	CGG GTG AAC GTG GAG AGA CA	GTC CAG GGT TGC CAT TGG AG
Decorin	ACC TTC ACA ACA ACA ATA TCT CTG	AGC ACG CAC ATA GAC ACA TC
Elastin	TTG GCG GCT TAG GAG TCT C	GCA CTT TCC CAG GCT TCA C
Lumican	AGC ACC TAT CCT GAT TAC TAT GAG	GTC AAT CTG GTT ATT CCG AAG G
Versican	CTG GAG AAG ATT GTG TTG	GTG TAG GTG AGA TGG TAA
ADAMTS-4	TCC TGG CTG GCT TCC TCT TC	CCT CGG ACA AGT CTT CAG AAT CTC
MMP-3	CTT CCG ATT CTG CTG TTG CTA TG	ATG GTG TCT TCC TTG TCC CTT G
MMP-13	TCC TGG CTG GCT TCC TCT TC	CCT CGG ACA AGT CTT CAG AAT CTC
TIMP-1	CAA CTC CGA TGT CGT CAT	TCT CAT AAC GCT GGT ATA AGG
TIMP-2	TTG GAG GAA AGA AGG AGT A	CAC GAT GAA GTC ACA GAG
TIMP-3	CAG CAG ATA GAC TCA AGG T	GAC ACA GAC AGA CAC AGT

ADAMTS-4 (a disintegrin and metalloproteinase with thrombospondin motifs 4), MMP-3 and MMP-13 (matrix metalloproteinase-3, 13), TIMP (tissue inhibitor of metalloproteinase).

### Caspase 3/7 Activity

Caspase 3/7 activity of the disc was determined as previously described [38]. Briefly, the disc tissues were pulverized with a mortar and pestle and homogenized with the polytron mixer (Kinematica, USA) in lysis buffer consisting of 25 nM HEPES (pH 7.5), 5 mM MgCl_2,_ 5 nM dithiotritol (DDT), 1 mM phenylmethanesulfonylfluoride (PMSF), 1 µg/mL benzamidine (Sigma-Aldrich, Buchs, Switzerland). One hundred µL of the supernatant was transferred into a 96-well plate and the Caspase Glo 3/7 assay was performed according to the manufacturer's protocol (Promega, Wallisellen, Switzerland). The generated luminescence was detected after 1 h incubation using a microplate reader (SpectraMax M5, Molecular device, Switzerland). Total protein concentration of the samples was quantified according to Bradford’s method [39]. The caspase 3/7 activities of the samples were normalized to the total protein content obtained from the Bradford assay.

### Quantification of GAG, HYP and DNA Content

Tissues from the resazurin red assay were dried at 60°C overnight and then digested with papain in buffer containing 55 mM sodium citrate, 150 mM sodium chloride, 5 mM EDTA and 5 mM cysteine hydrochloride, pH 6.0, (Sigma-Aldrich, Buchs, Switzerland) overnight at 60°C. The papain-digested samples were used for glycosaminoglycan (GAG) and DNA measurement. The GAG content was measured by the modified dimethyl-methylene blue (DMB) assay (pH 1.5) [40]. The absorbance of the samples added to the DMB buffer was read at 595 nm with a microplate reader (SpectraMax M5, Molecular device. Switzerland). Part of the papain-digested samples were hydrolyzed with 6 M HCl at 96°C for 24 hours to release hydroxyproline (HYP). HYP content was determined as a measure of total collagen content. Color reaction was prepared using chloramine T and 4-dimethyl-amino-benzaldehyd (DABA) (Sigma-Aldrich, Buchs, Switzerland), then absorbance was read at 560 nm. The amount of DNA in the sample was measured with bisbenzimidol fluorescent dye (Hoechst 33258, Sigma-Aldrich) and read with a microplate reader (SpectraMax M5, Molecular device, Switzerland). GAG, HYP and DNA concentrations were calculated from a standard curve obtained with chondroitin sulfate, HYP and calf thymus DNA (all from Sigma-Aldrich, Buchs, Switzerland) respectively.

### Histology

On the day of harvesting (day 0 control) and after the 15 days of culturing (four loading samples), discs were harvested and the disc was cut in the sagittal plane into two halves retaining the endplates (N = 2). The disc was fixed in 4% paraformaldehyde (PFA) and sequentially dehydrated and embedded in methyl methacrylate (MMA) without decalcification. Serial sections were cut in the sagittal plane at 6 µm with a microtome (Microm International, Waldorf, Germany). Staining was carried out with safranin O & fast green to show the overall matrix organization and cell morphology. High-resolution photos were taken with a digital camera (Eclipse 800, Nikon, Tokyo, Japan).

### Statistics

The statistical analysis was performed using the PRISM software (GraphPad, La Jolla, USA). A significance value of p<0.05 was specified to be significant. Two-way ANOVA with Turkey’s multiple comparison test was used for all the analyses, except gene expression analysis, which was performed by Wilcoxon signed-rank test compared to a theoretical mean of 1.

## Results

### Changes in Dimensions

**Figure 3:**
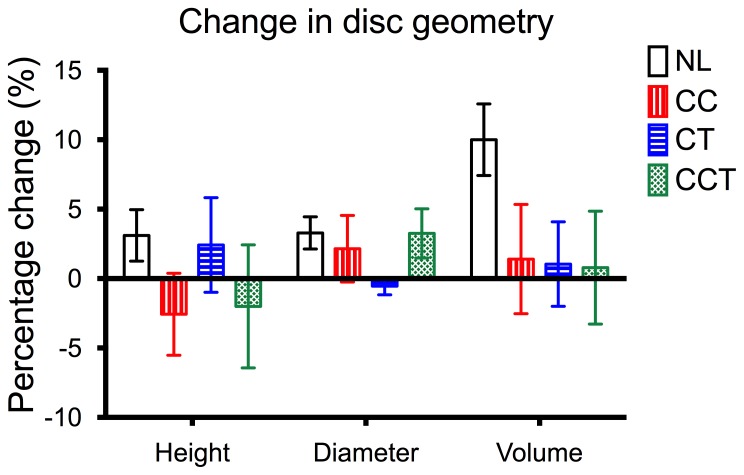
Percentage change in disc dimension relative to the starting of the experiment. There was a 10% increase in disc volume in the No loading (NL) group, but increase was minor in all the other 3 groups. CC: cyclic compression, CT: cyclic torsion, CCT: combined compression torsion. Plot of means ± SEM. N = 6.

Discs used in this study had a mean dimension of 16.63±1.55 mm diameter and 9.58±1.22 mm height at day 0. By the end of the experiment, disc volume was increased by 10±5.76% for NL, but increase in disc volume were less than 2% in all the other groups with loading (Figure 3). There was a slight increase in mean disc height of around 3% in the NL and CT groups, while disc height was decreased by about 2% in the groups with cyclic compression (CC and CCT). There was a 2–3% increase in disc diameter for all groups except for CT, where rotation was applied with a 0.2 MPa static compression, in which disc diameter decreased by 0.5% (Figure 3).

### Cell Viability and Activity

**Figure 4:**
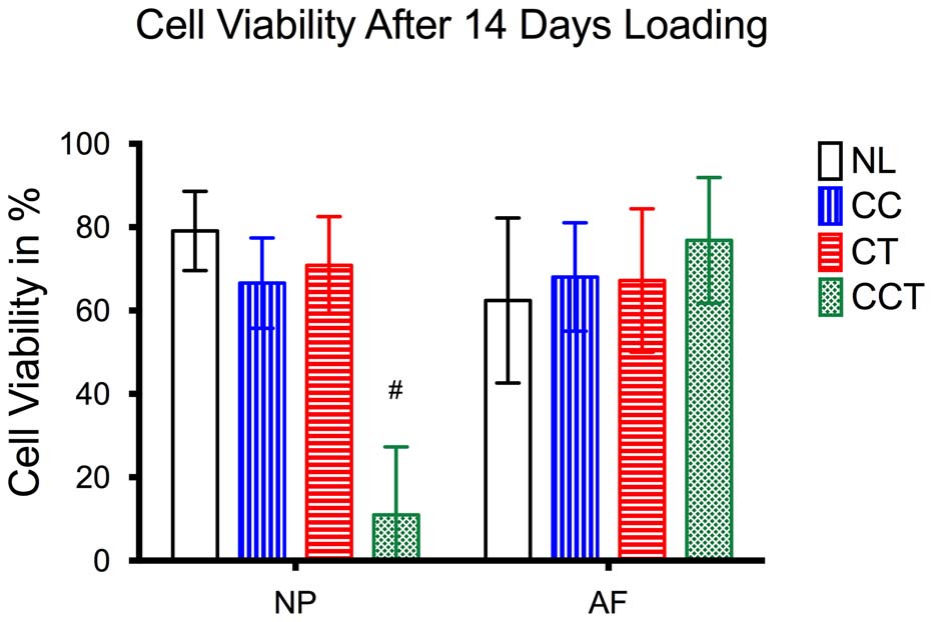
Percentage cell viability of the disc tissue measured by fluorescence live/dead stain and confocal microscopy. There was a significant cell death in the nucleus pulposus (NP) of the combined compression torsion (CCT) group compared to no loading (NL), cyclic compression (CC) and cyclic torsion (CT) groups. Cell viability in the annulus fibrosus (AF) maintained stable for all groups. Plot of means ± SEM. # Statistically significant different from all the other groups, p<0.0001. N = 6.

**Figure 5:**
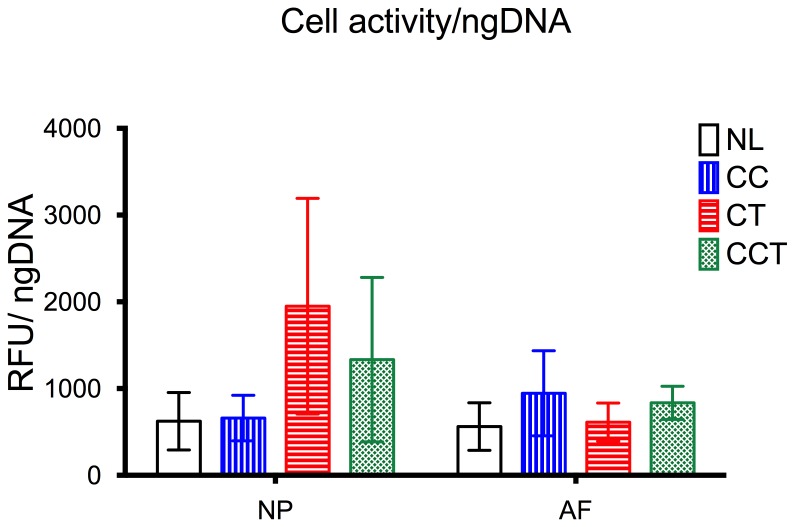
Activity of the cells measured by resazurin assay. Cell activity was doubled in the NP of groups with torsion (CT and CCT) compared to when torsion was not applied (NL and CC). NL: no loading, CC: cyclic compression, CT: cyclic torsion, CCT: combined compression torsion, NP: nucleus pulposus, AF: annulus fibrosus. Plot of means ± SEM. N = 6.

Disc cell viability was maintained at 62% in the AF and 79.09±9.49% in the NP when no loading was applied (NL). Two-way ANOVA showed statistically a significant difference in cell viability for both loading methods (p = 0.001) and region of the disc tissue (p = 0.003). Cell viability in the NP of the CCT group dropped to 10.99±16.3%, which was significantly lower than the cell viability in the NP of all the other three groups and in the AF of all groups (post-hoc multiple comparison all p<0.0001). In the AF, the cell viability of the CCT group (76.83±15.07%) was almost 15% higher than in NL (62.42±19.79%) (Figure 4). Two-way ANOVA showed a statistically significant difference in the cell activity, analyzed by resazurin assay with the different loading patterns (p = 0.048) (Figure 5).

### Caspase 3/7 Content

**Figure 6:**
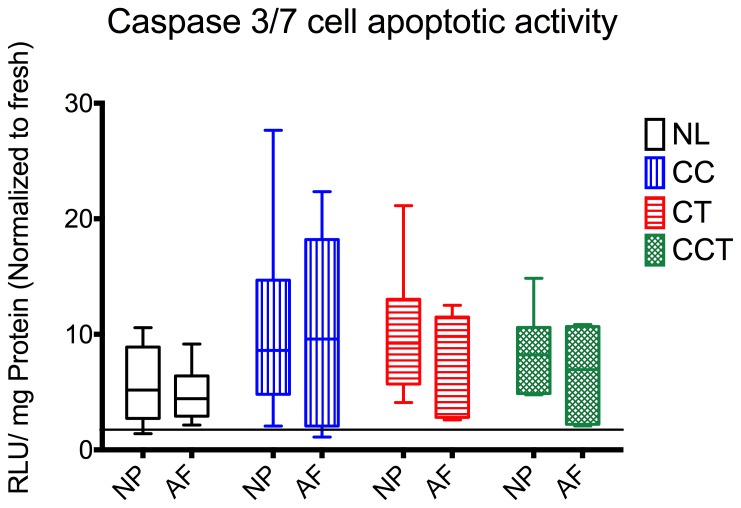
Cell apoptotic activity quantified by caspase 3/7 protein normalized by the total protein content. No statistically significant difference in caspase 3/7 protein amount was found between groups. NL: no loading, CC: cyclic compression, CT: cyclic torsion, CCT: combined compression torsion, NP: nucleus pulposus, AF: annulus fibrosus. Plot of means ± SEM. * statistically significant up-regulation p<0.05. N = 6.

Cell apoptotic activities were all increased over the 15 days culture time (p = 0.022), however there were no statistically significant differences in caspase 3/7 protein between different groups or different region of the disc (Figure 6).

### Real-time RT-PCR

**Figure 7:**
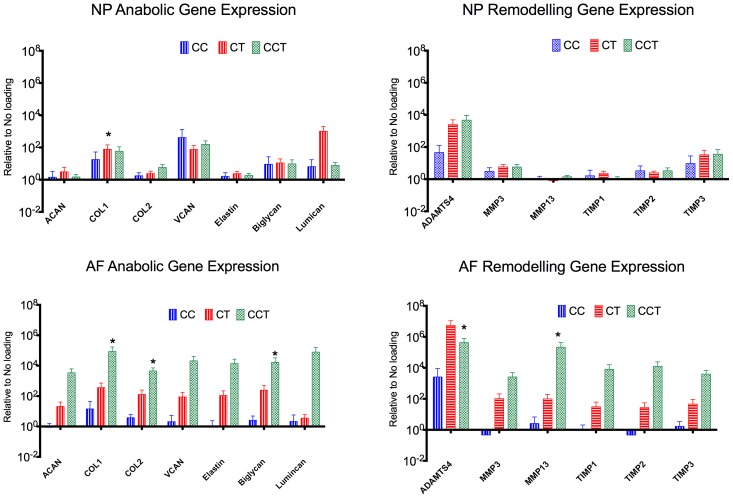
Relative gene expression quantified by real-time PCR. A group of anabolic genes and remodeling genes were analyzed in the NP and AF tissues. Both anabolic genes and remodeling genes were activated in the AF of both CT and CCT when torsion was applied. Plot of log means ± SEM, normalized to no loading control. * p<0.05. NL: no loading, CC: cyclic compression, CT: cyclic torsion, CCT: combined compression torsion, NP: nucleus pulposus, AF: annulus fibrosus. N = 6.G.

A group of anabolic genes (collagen 1 [COL1A2], collagen 2 [COL2A1], proteoglycans: aggrecan [ACAN], versican [VCAN], biglycan, lumican and elastin) and remodeling genes (ADAMTS-4, MMP-3, -13, TIMP-1, 2, 3) were analyzed. Changes in both anabolic and remodeling gene expressions were rather mild in the NP, as compared to the AF. In the NP, collagen 1 expression was significantly up-regulated in CT (p = 0.031). ADAMTS-4 was increased over 1000 fold in both CT and CCT, where its inhibitor TIMP-3 was also increased more than 10-fold (Figure 7).

In the AF of the CCT group, anabolic genes including collagen 1, collagen 2 and biglycans were significantly increased, the increase in the catabolic genes ADAMTS-4 and MMP-13 were also statistically significant. There was also a trend of mild increase in gene expression levels in CC that were less than 10-fold increased in all the analyzed genes. In CT, where torsion was present, most genes were up-regulated by 100-fold. In CCT where both dynamic compression and torsion were present, all genes were up-regulated by at least 1,000- to 100,000-fold (Figure 7).

### GAG and HYP Content

**Figure 8:**
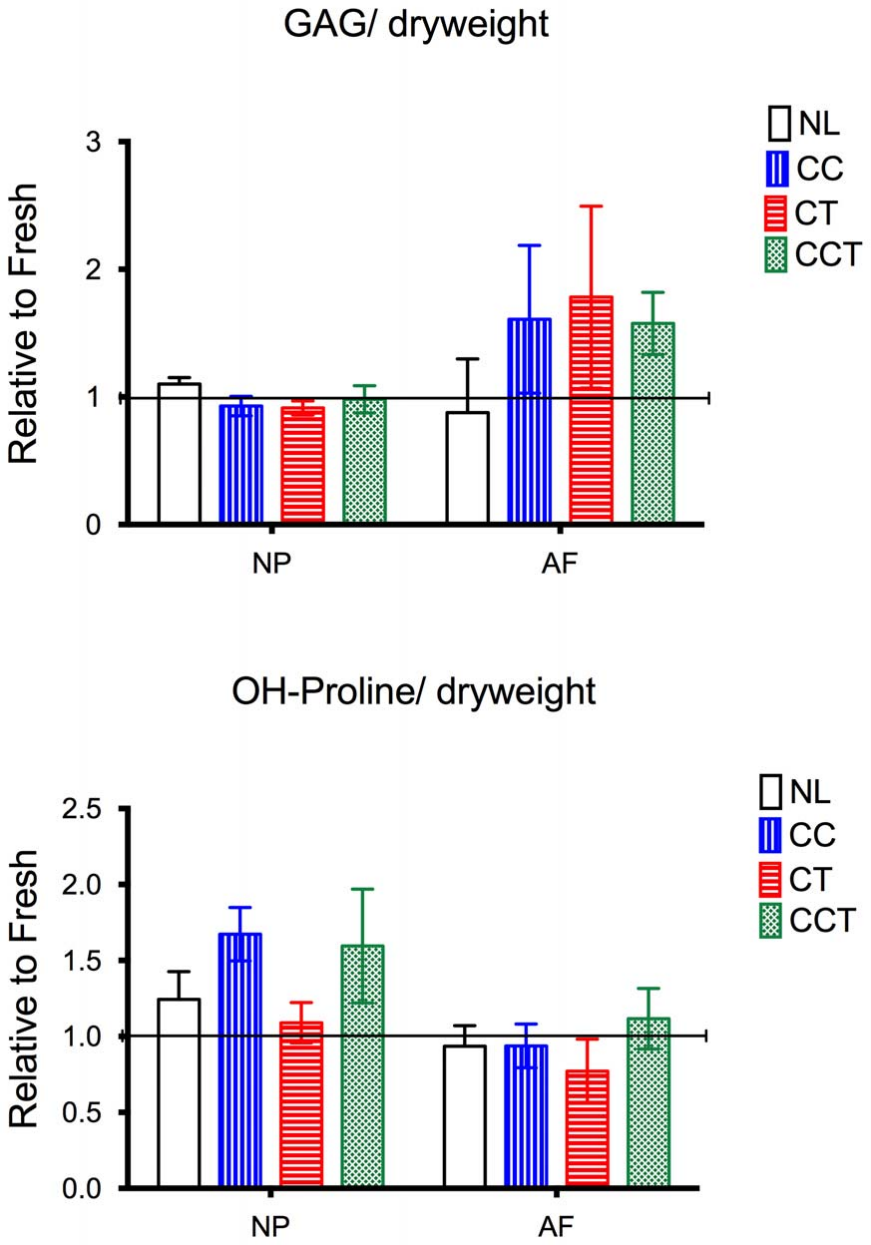
Glycosaminoglycans (GAG) and hydroxyproline (HYP) contents of the tissue normalized to the fresh samples. GAG content was maintained in all samples with a minor increase in the AF of all the 3 loading groups. HYP content was maintained without significant change. NL: no loading, CC: cyclic compression, CT: cyclic torsion, CCT: combined compression torsion, NP: nucleus pulposus, AF: annulus fibrosus. Plot of means ± SEM. N = 6.

Both GAG and HYP content of the disc tissues showed no change during the culture period (Figure 8). GAG content ratio relative to day 0 was close to 1 in the NP of all groups. There was even a tendency to a slight increase in mean GAG content in the AF of the CCT groups (1.482, p = 0.065) (Figure 8 upper).

### Histology

**Figure 9:**
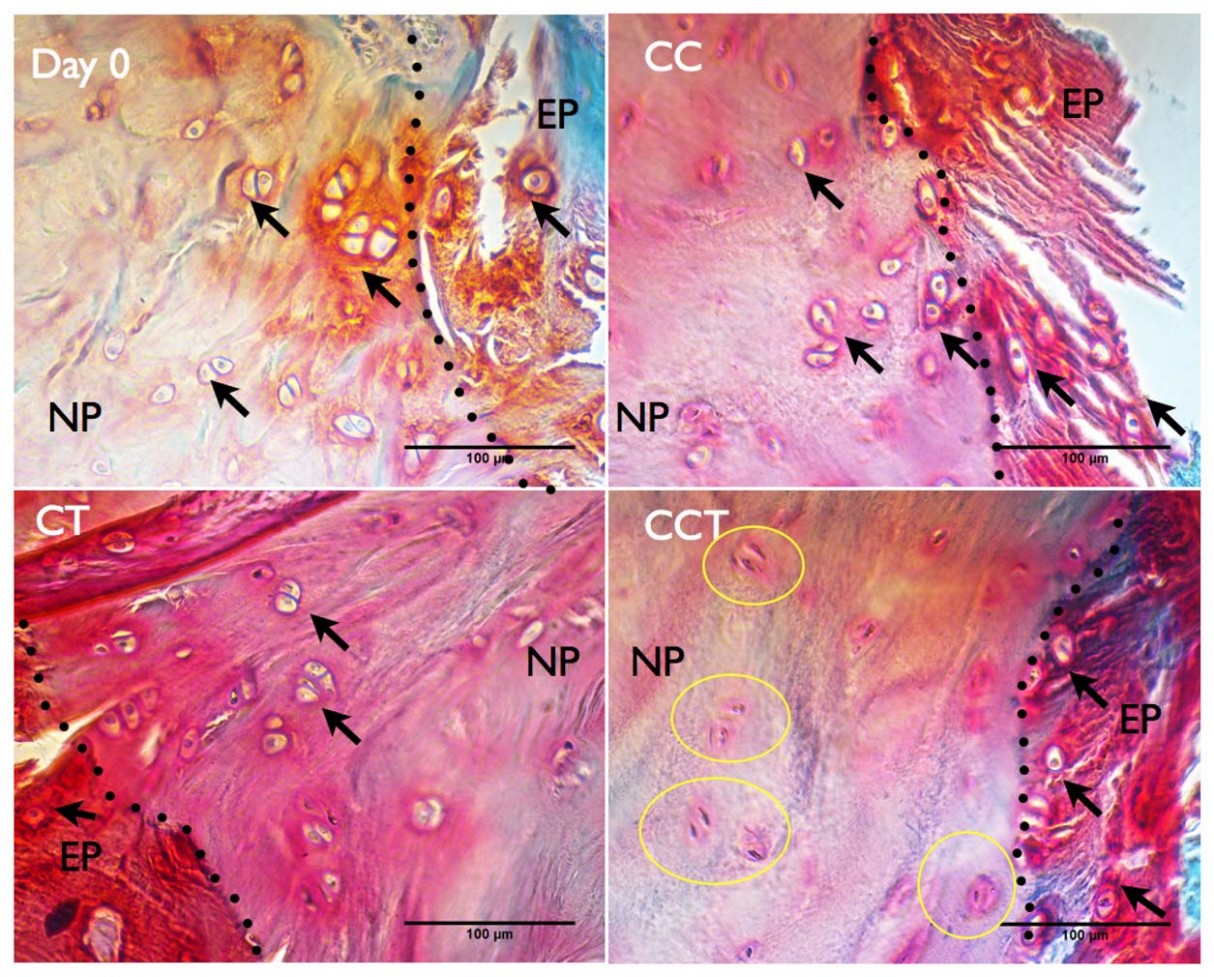
Histological analysis showing morphology of cells in the transition zone of the endplate and the nucleus pulposus. MMA sections were stained with safranin O/fast green. Cells in the transition zone between the cartilaginous endplates (EP) and nucleus pulposus (NP) (separated by dotted black line) stayed as rounded chondrocyte-like cells (indicated by black arrows) as in hyaline cartilage in CC and CT groups. Some cells in the cartilaginous endplates of the CCT group stayed as chondrocyte-like cells whereas cells right after the endplates zone changed to cells with spindle-shaped nucleus and cell lacunae were lost (indicated by yellow circles). CC: cyclic compression, CT: cyclic torsion, CCT: combined compression torsion. N = 2.

Histological sections were stained with safranin O & fast green to show the morphology of the cells and the overall matrix organization. No significant changes in matrix organization between different groups could be observed (data not shown). In the transition zone between the cartilaginous endplates (EP) and the nucleus pulposus (NP) (Figure 9, EP/NP border indicated by dotted black line), cells stayed as chondrocyte-like cells (indicated by black arrows) in the CC and CT groups with a round cell nucleus surrounded by lacunae. However, in CCT, very few cells stayed as chondrocyte-like cells (indicated by black arrows) in the cartilaginous endplate and cells right across the endplate region changed to spindle-shaped (indicated by a yellow circle) and the cell lacunae and the cell boundary were lost.

## Discussion

This study compared three different loading modalities applied on disc explants with endplates using a newly designed dynamic bioreactor for combined compression and torsion of discs in organ culture. Since data on physiological loading magnitudes are incomplete for bovine, the loading magnitudes applied in this study were based on the physiological range of loading in humans: cyclic compression (CC) of 0. 6±0.2 MPa at 0.2 Hz [41] which has been used for other large animal disc culture studies [42], [43], cyclic torsion of ±2° at 0.2 Hz [17], [38], and combined compression and torsion (CCT) was the combination of axial compression (CC) and axial torsion (CT). In our previous experiment, we tested the influence of increasing the torsion angle (0°, ±2°, ±5°, ±10°) using bovine caudal discs and found that a torsion angle>±5° could be harmful to the disc and ±2° dynamic torsion applied for 1 h/day in the short term even increased cell viability in organ culture [38]. To further support the validity of using bovine disc samples for this mechano-biological study, we have also compared the torsional stiffness of the bovine motion segment, supporting that bovine discs have a similar torsional stiffness to human cervical discs [38]. Other studies also provided data supporting the use of bovine caudal discs as a valid model for comparing the torsional behavior of human discs [44] and for biological studies [45], [46].

Our results provided evidence that disc cells respond differently when subjected to complex dynamic torsion-compression loading, compared to simple dynamic loading. In the group of CCT, even though both compression and torsion were applied at their respective physiologic level, and have been shown not to induce disc degeneration when applied separately [38], [43], most of the NP cells were eradicated (<10% cell viability, Figure 4) during the 14 days of loading. Cell death could be caused by either apoptosis or necrosis. Caspase 3/7 proteins, which are a family of cysteine proteases that play essential roles in apoptosis, were measured in both NP and AF tissue, but no significant increase was found in the NP of CCT as compared to other experimental groups. The non-significant increase in caspase proteins implies that cell death in the NP might be caused through the caspase 3/7 independent pathways or by necrosis. We speculate that the high rate of cell death in the NP of the CCT group was caused by mechanical injury due to the shear stress that was applied to the NP cells; the random, non-oriented NP matrix was not able to protect cells from high shear force. Histological results also showed necrotic cell morphology of the cells in the NP of CCT indicating the possible cell damage due to trauma (Figure 9). In theory, torsion at low magnitude may induce stress to the AF but decompress the NP [47]. However, the presence of torsion during compression might result in a higher shear stress in the NP than compression without torsion. Furthermore, the hoop stresses induced by torsion add to those induced by compression and exerts a larger net stress on the NP matrix and cells, than simple compression or simple torsion. This sum of stresses may have exceeded the tolerance limit of the NP cells, thus causing a cell death in the NP of CCT. Kim et al. suggested that a shear force of 0.33 MPa was enough to cause disc degeneration, which was lower than the 0.8 MPa compression stress as suggested in other studies [48]. The magnitude of the shear force applied in CCT, however, was unknown. Other biomechanical studies have been performed to evaluate the influence of torsion on the disc and results indicated that torsion alone or combined with bending exerts shear stress to the disc and may cause failure in the annulus. Unfortunately, not much information is known about what happens in the NP during torsion-compression. Future in-silico studies may be performed to evaluate the shear stress that is applied to the NP during combined dynamic torsion-compression loading. In-vitro studies can also compare the influence of shear on NP and AF cells and the tolerance limits of disc cells to shear.

Although CCT has significantly reduced the cell viability in the NP, AF cell viability remained comparable to cyclic compression (CC) or cyclic torsion (CT) (>70% cell viability, Figure 4). AF cells were actively participating in matrix remodeling, as shown by a large increase in both anabolic and remodeling gene expression (Figure 7). We suspect that torsion-compression loading has caused micro-damage to the collagen, therefore disc cells have been activated to compensate for the destruction. As shown in the gene expression result, groups with torsion (CT and CCT) showed a larger increase in both anabolic and catabolic gene expression by AF cells as compared to no loading or pure compression, indicating that AF cells were more sensitive to torsional loading stimulation. Therefore they responded by increasing some matrix production and matrix destruction enzymes to remodel the matrix environment. These increases were also stronger in CCT than CT, which could be associated with an increased stimulus by CCT. Anabolic genes and remodeling genes were all increased by about 1,000 fold in the AF when torsion was applied with a 0.2 MPa static compression (CT). When torsion was applied together with a dynamic compression of 0.6±0.2 MPa (CCT), which represents a higher resultant shear stress applied on the whole organ, anabolic and remodeling gene expression were up-regulated by 1000–100,000 fold. In the NP, no striking changes could be seen in the analyzed genes except that ADAMTS-4 was increased by 100,000 fold in the NP of the groups with torsion (CT and CCT). This is in agreement with a previous study by Barbir *et al.* in which torsion has caused an increased ADAMTS-4 gene expression in the NP [20]. Barbir et al. also found a significant increase in elastin gene expression in their in-vivo study. Although elastin was up-regulated by 100,000 fold in the AF of CCT group, it was not statistically significant (p = 0.35).

One possible reason for the difference in response between the NP and AF to the same loading is due to the fundamental difference in the matrix component and structure between NP and AF. AF collagen fibers are aligned in an angle that can withstand shear force but the disorganized gel-like matrix of the NP cannot withstand a high shear force under combined compression and torsion. The NP, which is mainly composed of water, proteoglycans and collagen 2, is more resistant to compressive force than direct shear force as in compression and torsional load. A uniform torque applied to the disc will result in a hoop strain within the tissue, which increases with the distance from the center of rotation. It might be that the reaction of the annulus cells to the applied torsion stress is also different between the outer annulus and the inner annulus fibrosus as the inter-lamellar angle decreases radially from the periphery to the center from 60° towards 40°. Moreover, the elastic fiber arrangements in intra-lamellar and interlamellar zones were shown to be architecturally distinct, suggesting that they perform multiple functional roles within the AF matrix structural hierarchy [49]. Elastic fiber density was found to be significantly higher in the lamellae of the posterolateral regions of the AF than the anterolateral, and significantly higher in the outer regions than the inner, suggesting that elastic fiber density in each region of the annulus is commensurate with the magnitude of the tensile deformations experienced in bending and torsion [49]. An in vivo study also showed that cyclic torsion increased gene expression of elastin in the AF but not in the NP, indicating a possible strain magnitude dependent response [20]. Thus, application of two differently oriented stresses, compression and axial torsion, resulted in different biological responses of the AF and NP. There is evidence that AF cells in 3D culture are very sensitive to changes in mechanical load but this could not be shown in NP cells [50]. A study was performed using a finite element model to evaluate the effect of mechanical loading on the deformation of the intervertebral disc cells [51]. A larger deformation of the cells in the AF and the transition zone than in the NP was predicted. The AF cells might receive these mechanical signals and hence causing large increases in anabolic and remodeling gene expression so as to compensate or repair the matrix damaged by the shear loading. Another reason for this dramatically different response of NP cells relative to AF cells to a relatively physiological torsion could be the fact that the shape of the endplates of coccygeal discs facing the disc is concave in the sagittal direction rather than convex as in a human disc, thus the disc height is decreasing towards the center. This difference in shape of the endplates has been previously noted [52] and it has been recently suggested to have an influence on the distribution of the force between AF and NP in an uni-axial bioreactor system, where all remaining bone of the motional segments have been removed for culture except the cartilaginous endplates [53].

Pressure changes in the intervertebral disc are known to occur during compression [54]–[56], but little is known about the pressure changes during torsion. Van Deursen et al. (2001) [47] measured pressure changes with a transducer in pig intervertebral discs during torsion of 2° and noticed that there is a completely different behavior of pressure between the NP and the AF. The pressure in the nucleus rapidly decreased and the stress in the AF increased by 20–30%. In contrast, Yantzer et al. (2007) [57] found no significant difference in nuclear pressure when torsion was applied at different magnitudes ranging from 0–2 Nm. In theory, a pure axial compression of the disc results in a volumetric change and, consequently, a hydrostatic pressurization of the nucleus pulposus, accompanied by a moderate tensile stress in the annulus, which is required to contain the nuclear pressure. Torsion as pure shear is theoretically accompanied with no matrix strains and no volumetric strain for a homogeneous cylindrical material under small deformations; therefore it may be an approximate assumption for a cylindrical disc that no pressurization of the disc should be expected. This may not be an accurate assumption for a human lumbar disc. Depending on the fiber orientation of the annulus, a coupled height change in the disc during torsion may be induced by stretching and uncrimping of the collagen fiber in the annulus. Our result indicated a 2% increase in disc height in the group of CT (torsion with static compression) (Figure 3). Mechanically, collagen fibril sliding was demonstrated to govern cell mechanics and strain transfer in the AF during loading activities [58]. A recent clinical study performed using magnetic resonance imaging (MRI) also showed that during running exercise, disc height in the lumbar region, which involves mainly dynamic compression, was not changed after running; where in the thoracic region, which is a combined dynamic compression and rotation due to the motion of the upper body, disc height was significantly reduced after running [59]. As opposed to the previous study, our study found that disc height was decreased for around 2% in both groups with dynamic compression at a physiological range (0.6±0.2 MPa) as compared to the initial disc height. The co-existence of torsion and dynamic compression (CCT) did not seem to cause a larger decrease in disc height as compared to pure dynamic compression (CC), but torsion with a static compression of 0.2 MPa (CT) has caused a slight increase (2%) in disc height (Figure 3). In our experimental setup, we assume that the pressures in the three loading scenarios are equally distributed in the NP and AF. The changes in disc height are probably due to the difference in the applied pressure, which was lower in CT (0.2 MPa) than CC and CCT (0.6±0.2 MPa). The non-significant difference in the disc height between CC and CCT also implied a comparable pressure between CC and CCT, although there is some difference in stress and pressure between CC and CCT due to the presence of torsion in CCT.

In a cadaveric study of the mechanical behavior of the spine during torsion, it was proposed that torsion is unimportant in the etiology of disc degeneration and prolapse, since the interspinous ligament and the facet joint protect the disc from excessive axial rotation [60]. However, disc herniation has frequently appeared in athletes who have been participating in sports involving frequent torsional movement of the spine [1], [61]. Previous biomechanical studies also demonstrated that, combined torsion and compression increased the susceptibility to disc injury [25], [30], combined repetitive flexion-extension and compression also increased the chance of disc herniation in porcine specimens [24]. Repetitive torsional exertions imposed greater loading on the spine than tasks of lateral bending or flexion [62]. Our histological results also agree with the suggestion by Adams and Hutton (1981) [60] that torsion is unlikely to play a role in the mechanical disruption of the discs at physiological levels, but the biological influences of torsion-compression on the disc cells might affect the disc properties in the long term. Combined compression and torsion, even in a physiological range, can cause progressive injury to the disc on the biological level even without obvious mechanical damage to the disc structure. Although the AF is actively involved in remodeling the annulus in response to the loading, the altered cell phenotype and traumatic cell death in the NP could ultimately cause progressive disc degeneration in the long term [63].

There are a few in-vitro organ culture systems coupled with dynamic mechanical loading functions available for performing mechano-biological studies on disc explants. These systems usually can apply axial compression of different magnitude and frequency to large animal disc explants and have provided important information on the mechanobiology of the disc [43], [64], [65]. Although complex loading can mimic more closely the in-vivo loading [66], developing a bioreactor system for disc explants has faced some hurdles, especially in maintaining the viability of the organ in-vitro [43], [67]. Hartman and colleagues (2011) [68] have developed a bioreactor with 6-degree of freedom (DOF) load application for rabbit functional spinal units, but disc cell viability could be maintained for less than 30 h. We suspect that the presence of vertebra bone on both sides of the disc might limit nutrient perfusion into the disc therefore disc cell viability could not be maintained in this kind of system. Therefore, we have developed the described loading device for disc explants without vertebra bone but with the bony endplates [34]. We successfully applied both dynamic compression and torsion on the disc explants with preserved cell viability for up to 15 days. Future bioreactor designs should aim to incorporate a 6-DOF loading system for disc explants which maintain only enough vertebral endplate for stability.

## Conclusion

In conclusion, there is a region-specific response of disc cells to complex loading consisting of both dynamic compression and torsion. While complex loading causes traumatic cell death in the NP, the AF resists the high shear stress and responds by active matrix remodeling. This complex loading bioreactor can be used for the development of tissue-engineering disc construct to promote maturation of a construct matrix of interest under a physiological simulated mechanical environment closely related to the in-vivo situation [69], [70].
